# About the sharpness of the Jensen inequality

**DOI:** 10.1186/s13660-018-1923-4

**Published:** 2018-12-10

**Authors:** Ðilda Pečarić, Josip Pečarić, Mirna Rodić

**Affiliations:** 1 Catholic University of Croatia, Zagreb, Croatia; 2 Faculty of Textile Technology, University of Zagreb, Zagreb, Croatia; 3 RUDN University, Moscow, Russia

**Keywords:** 26D15, 60E05, 94A17, Green function, Jensen’s inequality, *f*-divergence, Entropy, Zipf–Mandelbrot law

## Abstract

The main aim of this paper is to give an improvement of the recent result on the sharpness of the Jensen inequality. The results given here are obtained using different Green functions and considering the case of the real Stieltjes measure, not necessarily positive. Finally, some applications involving various types of *f*-divergences and Zipf–Mandelbrot law are presented.

## Introduction

The Jensen inequality is one of the most famous and most important inequalities in mathematical analysis.

In [2], some estimates about the sharpness of the Jensen inequality are given. In particular, the difference

$$ \int_{0}^{1} \varphi \bigl( f(x) \bigr) \,dx - \varphi \biggl( \int_{0} ^{1} f(x) \,dx \biggr) $$

is estimated, where *φ* is a convex function of class $C^{2}$.

The authors in [2] expanded $\varphi ( f(x) ) $ around any given value of $f(x)$, say around $c=f(x_{0})$, which can be arbitrarily chosen in the domain *I* of *φ*, such that $c=f(x_{0})$ is in the interior of *I*, and as their first result, they get the following inequalities:

$$\begin{aligned} 0 & \leq \int_{0}^{1} \varphi \bigl( f(x) \bigr) \,dx - \varphi \biggl( \int _{0}^{1} f(x) \,dx \biggr) \\ & \leq \frac{1}{2} \bigl\Vert \varphi '' \bigr\Vert _{L^{\infty }(I_{2})} \cdot \Vert f-c \Vert _{L^{2}}^{2} + \frac{1}{2} \bigl\Vert \varphi '' \bigr\Vert _{L^{\infty }(I_{2})} \cdot \Vert f-c \Vert _{L^{1}}^{2} \\ & = \frac{1}{2} \bigl\Vert \varphi '' \bigr\Vert _{L^{\infty }(I_{2})} \cdot \bigl[ \Vert f-c \Vert _{L^{2}}^{2} + \Vert f-c \Vert _{L^{1}}^{2} \bigr] , \end{aligned}$$

where $I_{2}$ denotes the domain of $\varphi ''$.

The main aim of our paper is to give an improvement of that result using various Green functions and considering the case of the real Stieltjes measure, not necessarily positive.

## Preliminary results

Consider the Green functions $G_{k}:[\alpha , \beta ]\times [\alpha , \beta ]\rightarrow \mathbb{R}$ ($k=0,1,2,3,4$) defined by

1 $$\begin{aligned}& G_{0}(t,s)= \textstyle\begin{cases} \frac{(t-\beta )(s-\alpha )}{\beta -\alpha } & \text{for } \alpha \leq s\leq t, \\ \frac{(s-\beta )(t-\alpha )}{\beta -\alpha } & \text{for } t\leq s \leq \beta , \end{cases}\displaystyle \end{aligned}$$

2 $$\begin{aligned}& G_{1}(t,s)= \textstyle\begin{cases} \alpha -s & \text{for } \alpha \leq s\leq t, \\ \alpha -t & \text{for } t\leq s \leq \beta , \end{cases}\displaystyle \end{aligned}$$

3 $$\begin{aligned}& G_{2}(t,s)= \textstyle\begin{cases} t-\beta & \text{for } \alpha \leq s\leq t, \\ s-\beta & \text{for } t\leq s \leq \beta , \end{cases}\displaystyle \end{aligned}$$

4 $$\begin{aligned}& G_{3}(t,s)= \textstyle\begin{cases} t-\alpha & \text{for } \alpha \leq s\leq t, \\ s-\alpha & \text{for } t\leq s \leq \beta , \end{cases}\displaystyle \end{aligned}$$

5 $$\begin{aligned}& G_{4}(t,s)= \textstyle\begin{cases} \beta -s & \text{for } \alpha \leq s\leq t, \\ \beta -t & \text{for } t\leq s \leq \beta . \end{cases}\displaystyle \end{aligned}$$

All these functions are convex and continuous with respect to both *s* and *t*.

We have the following lemma (see also [16] and [17]).

### Lemma 1

*For every function* $\varphi \in C^{2}[\alpha , \beta ]$, *we have the following identities*:

6 $$\begin{aligned}& \varphi (x)=\frac{\beta -x}{\beta -\alpha }\varphi (\alpha )+\frac{x- \alpha }{\beta -\alpha } \varphi (\beta )+ \int_{\alpha }^{\beta }G_{0}(x,s) \varphi ''(s)\,ds, \end{aligned}$$

7 $$\begin{aligned}& \varphi (x)=\varphi (\alpha )+(x-\alpha )\varphi '( \beta )+ \int_{ \alpha }^{\beta }G_{1}(x,s)\varphi ''(s)\,ds, \end{aligned}$$

8 $$\begin{aligned}& \varphi (x)=\varphi (\beta )+(x-\beta )\varphi '( \alpha )+ \int_{ \alpha }^{\beta }G_{2}(x,s)\varphi ''(s)\,ds, \end{aligned}$$

9 $$\begin{aligned}& \varphi (x)=\varphi (\beta )-(\beta -\alpha )\varphi '(\beta )+ (x- \alpha )\varphi '(\alpha ) + \int_{\alpha }^{\beta }G_{3}(x,s)\varphi ''(s)\,ds, \end{aligned}$$

10 $$\begin{aligned}& \varphi (x)=\varphi (\alpha )+(\beta -\alpha )\varphi '(\alpha )- ( \beta -x)\varphi '(\beta ) + \int_{\alpha }^{\beta }G_{4}(x,s)\varphi ''(s)\,ds, \end{aligned}$$

*where the functions* $G_{k}$ ($k=0,1,2,3,4$) *are defined as before in* (1)*–*(5).

### Proof

By integrating by parts we get

$$\begin{aligned} \int_{\alpha }^{\beta }G_{0}(x,s)\varphi ''(s)\,ds & = \int_{\alpha } ^{x} G_{0}(x,s)\varphi ''(s)\,ds + \int_{x}^{\beta }G_{0}(x,s)\varphi ''(s)\,ds \\ & = \int_{\alpha }^{x} \frac{(x-\beta )(s-\alpha )}{\beta -\alpha } \varphi ''(s)\,ds + \int_{x}^{\beta }\frac{(s-\beta )(x-\alpha )}{ \beta -\alpha } \varphi ''(s)\,ds \\ & = \frac{x-\beta }{\beta -\alpha } \int_{\alpha }^{x}(s-\alpha ) \varphi ''(s) \,ds + \frac{x-\alpha }{\beta -\alpha } \int_{x}^{\beta }(s- \beta ) \varphi ''(s) \,ds \\ & = \varphi (x)- \frac{\beta - x}{\beta -\alpha } \varphi (\alpha )- \frac{x-\alpha }{\beta -\alpha }\varphi (\beta ). \end{aligned}$$

The other identities are proved analogously. □

### Remark 1

The result (7) given in the previous lemma represents a special case of the representation of the function *φ* using the so-called “two-point right focal” interpolating polynomial in the case where $n = 2$ and $p = 0$ (see [1]).

Using the results from the previous lemma, the authors in [16] and [17] gave the uniform treatment of the Jensen-type inequalities, allowing the measure also to be negative. In this paper, we give some further interesting results.

## Main results

To simplify the notation, for functions *g* and *λ*, we denote

$$ \overline{g}=\frac{\int_{a}^{b} g(x)\,d\lambda (x)}{\int_{a}^{b} \,d \lambda (x)}. $$

### Theorem 1

*Let* $g:[a,b]\rightarrow \mathbb{R}$ *be a continuous function*, *and let* $\varphi \in C^{2}[\alpha , \beta ]$, *where the image of* *g* *is a subset of* $[\alpha , \beta ]$. *Let* $\lambda :[a,b]\rightarrow \mathbb{R}$ *be a continuous function or a function of bounded variation such that* $\lambda (a)\neq \lambda (b)$ *and* $\overline{g}\in [\alpha , \beta ]$. *Let the functions* $G_{k}: [\alpha , \beta ]\times [\alpha , \beta ]\rightarrow \mathbb{R}$ ($k=0,1,2,3,4$) *be as defined in* (1)*–*(5). *Let* $p,q\in \mathbb{R}$, $1\leq p,q \leq \infty $, *be such that* $\frac{1}{p}+\frac{1}{q}=1$.

*Then*

11 $$ \biggl\vert \frac{\int_{a}^{b} \varphi ( g(x) ) \,d\lambda (x)}{\int _{a}^{b} \,d\lambda (x)}-\varphi ( \overline{g} ) \biggr\vert \leq Q \cdot \bigl\Vert \varphi '' \bigr\Vert _{p}, $$

*where*

12 $$ Q= \textstyle\begin{cases} [ \int_{\alpha }^{\beta } \vert \frac{\int_{a}^{b} G_{k} ( g(x),s ) \,d\lambda (x)}{\int_{a}^{b} \,d\lambda (x)}-G_{k} ( \overline{g},s ) \vert ^{q} \,ds ] ^{\frac{1}{q}} & \textit{for } q \neq \infty , \\ \sup_{s\in [\alpha ,\beta ]} \{ \vert \frac{\int_{a}^{b} G_{k} ( g(x),s ) \,d\lambda (x)}{\int_{a}^{b} \,d\lambda (x)}-G_{k} ( \overline{g},s ) \vert \} & \textit{for } q = \infty . \end{cases} $$

### Proof

From Lemma 1 we know that we can represent every function $\varphi \in C^{2}[\alpha , \beta ]$ in adequate form using the previously defined functions $G_{k}$ ($k=0,1,2,3,4$). By some calculation we can easily get that, for every function $\varphi \in C^{2}[\alpha , \beta ]$ and for any $k\in \{0,1,2,3,4\}$, we have

13 $$\begin{aligned} \frac{\int_{a}^{b} \varphi ( g(x) ) \,d\lambda (x)}{\int_{a} ^{b} \,d\lambda (x)}- \varphi ( \overline{g} ) = \int_{\alpha } ^{\beta } \biggl[ \frac{\int_{a}^{b} G_{k} ( g(x),s ) \,d\lambda (x)}{ \int_{a}^{b} \,d\lambda (x)}- G_{k} ( \overline{g},s ) \biggr] \varphi ''(s) \,ds. \end{aligned}$$

Taking the absolute value to (13), using the triangle inequality for integrals, and then applying the Hölder inequality, we get:

$$\begin{aligned} & \biggl\vert \frac{\int_{a}^{b} \varphi ( g(x) ) \,d\lambda (x)}{\int _{a}^{b} \,d\lambda (x)}- \varphi ( \overline{g} ) \biggr\vert \\ &\quad = \biggl\vert \int_{\alpha }^{\beta } \biggl[ \frac{\int_{a}^{b} G_{k} ( g(x),s ) \,d\lambda (x)}{\int_{a}^{b} \,d\lambda (x)}- G_{k} ( \overline{g},s ) \biggr] \varphi ''(s) \,ds \biggr\vert \\ & \quad \leq \int_{\alpha }^{\beta } \biggl\vert \biggl[ \frac{\int_{a}^{b} G_{k} ( g(x),s ) \,d\lambda (x)}{\int_{a}^{b} \,d\lambda (x)}- G _{k} ( \overline{g},s ) \biggr] \varphi ''(s) \biggr\vert \,ds \\ & \quad \leq \biggl( \int_{\alpha }^{\beta } \biggl\vert \frac{\int_{a}^{b} G_{k} ( g(x),s ) \,d\lambda (x)}{\int_{a}^{b} \,d\lambda (x)}- G _{k} ( \overline{g},s ) \biggr\vert ^{q} \,ds \biggr) ^{\frac{1}{q}} \cdot \biggl( \int_{\alpha }^{\beta } \bigl\vert \varphi ''(s) \bigr\vert ^{p} \,ds \biggr) ^{\frac{1}{p}}, \end{aligned}$$

and the statement of the theorem follows. □

Now consider the case $q=1$, that is, $p=\infty $. If the positivity of the term $\frac{\int_{a}^{b} G_{k} ( g(x),s ) \,d\lambda (x)}{\int_{a} ^{b} \,d\lambda (x)}- G_{k} ( \overline{g},s ) $ does not change for all $s\in [\alpha , \beta ]$, then we can calculate *Q*. We have the following result.

### Corollary 1

*Let* $g:[a,b]\rightarrow \mathbb{R}$ *be a continuous function*, *and let* $\varphi \in C^{2}([\alpha , \beta ])$, *where the image of* *g* *is a subset of* $[\alpha , \beta ]$. *Let* $\lambda :[a,b]\rightarrow \mathbb{R}$ *be a continuous function or a function of bounded variation such that* $\lambda (a)\neq \lambda (b)$ *and* $\overline{g}\in [\alpha , \beta ]$. *Let the functions* $G_{k}: [\alpha , \beta ]\times [\alpha , \beta ]\rightarrow \mathbb{R}$ ($k=0,1,2,3,4$) *be as defined in* (1)*–*(5). *Suppose that*, *for any* $k\in \{0,1,2,3,4 \}$,

14 $$ \frac{\int_{a}^{b} G_{k} ( g(x),s ) \,d\lambda (x)}{\int_{a} ^{b} \,d\lambda (x)}-G_{k} ( \overline{g},s ) \geq 0 $$

*for all* $s\in [\alpha , \beta ]$ *or that*, *for any* $k\in \{0,1,2,3,4\}$, *the reverse inequality in* (14) *holds for all* $s\in [\alpha , \beta ]$. *Then*

15 $$ \biggl\vert \frac{\int_{a}^{b} \varphi ( g(x) ) \,d\lambda (x)}{\int _{a}^{b} \,d\lambda (x)}-\varphi ( \overline{g} ) \biggr\vert \leq \frac{1}{2} \cdot \bigl\Vert \varphi '' \bigr\Vert _{\infty }\cdot \biggl\vert \frac{ \int_{a}^{b} ( g(x) ) ^{2} \,d\lambda (x)}{\int_{a}^{b} \,d\lambda (x)}- ( \overline{g} ) ^{2} \biggr\vert . $$

### Proof

Let us start from the previous theorem and set $q=1$, $p=\infty $. Consider any $k\in \{0,1,2,3,4\}$. Then (11) transforms into

16 $$ \biggl\vert \frac{\int_{a}^{b} \varphi ( g(x) ) \,d\lambda (x)}{\int _{a}^{b} \,d\lambda (x)}-\varphi ( \overline{g} ) \biggr\vert \leq \bigl\Vert \varphi '' \bigr\Vert _{\infty }\cdot \int_{\alpha }^{\beta } \biggl\vert \frac{\int_{a}^{b} G_{k} ( g(x),s ) \,d\lambda (x)}{\int _{a}^{b} \,d\lambda (x)}- G_{k} ( \overline{g},s ) \biggr\vert \,ds. $$

When the positivity of the term $\frac{\int_{a}^{b} G_{k} ( g(x),s ) \,d\lambda (x)}{\int_{a}^{b} \,d\lambda (x)}- G_{k} ( \overline{g},s ) $ for all $s\in [\alpha , \beta ]$ does not change, we can calculate the integral on the right side of (16):

17 $$\begin{aligned} & \int_{\alpha }^{\beta } \biggl( \frac{\int_{a}^{b} G_{k} ( g(x),s ) \,d \lambda (x)}{\int_{a}^{b} \,d\lambda (x)}- G_{k} ( \overline{g},s ) \biggr) \,ds = \frac{1}{2} \cdot \biggl[ \frac{ \int_{a}^{b} ( g(x) ) ^{2} \,d\lambda (x)}{\int_{a}^{b} \,d\lambda (x)}- ( \overline{g} ) ^{2} \biggr] . \end{aligned}$$

(For the proof, see [16] and [17].)

If inequality (14) holds for all $s\in [\alpha , \beta ]$, then (16) becomes

18 $$ \biggl\vert \frac{\int_{a}^{b} \varphi ( g(x) ) \,d\lambda (x)}{\int _{a}^{b} \,d\lambda (x)}- \varphi ( \overline{g} ) \biggr\vert \leq \frac{1}{2} \cdot \bigl\Vert \varphi '' \bigr\Vert _{\infty }\cdot \biggl[ \frac{ \int_{a}^{b} ( g(x) ) ^{2} \,d\lambda (x)}{\int_{a}^{b} \,d\lambda (x)}- ( \overline{g} ) ^{2} \biggr] . $$

Also, if the reverse inequality in (14) holds for all $s\in [\alpha , \beta ]$, then (16) becomes

19 $$ \biggl\vert \frac{\int_{a}^{b} \varphi ( g(x) ) \,d\lambda (x)}{\int _{a}^{b} \,d\lambda (x)}-\varphi ( \overline{g} ) \biggr\vert \leq \frac{1}{2} \cdot \bigl\Vert \varphi '' \bigr\Vert _{\infty }\cdot \biggl[ ( \overline{g} ) ^{2} - \frac{\int_{a}^{b} ( g(x) ) ^{2} \,d\lambda (x)}{\int _{a}^{b} \,d\lambda (x)} \biggr] . $$

This means that if inequality (14) or the reverse inequality in (14) holds for all $s\in [\alpha , \beta ]$, then we have (15). □

### Remark 2

We can get the same result using the Lagrange mean-value theorems from [16] and [17], as they state that, for the functions *g*, *φ*, *λ*, $G_{k}$ ($k=0,1,2,3,4$) defined as in the previous theorem, if inequality (14) or the reverse inequality in (14) holds for all $s\in [\alpha , \beta ]$, then there exists $\xi \in [\alpha , \beta ]$ such that

20 $$\begin{aligned} \frac{\int_{a}^{b} \varphi ( g(x) ) \,d\lambda (x)}{\int_{a} ^{b} \,d\lambda (x)}- &\varphi ( \overline{g} ) = \frac{1}{2} \varphi ''(\xi ) \biggl[ \frac{\int_{a}^{b} ( g(x) ) ^{2} \,d \lambda (x)}{\int_{a}^{b} \,d\lambda (x)} - ( \overline{g} ) ^{2} \biggr] . \end{aligned}$$

The next result represents an improvement of the aforementioned result from [2].

### Corollary 2

*Let* $g:[a,b]\rightarrow \mathbb{R}$ *be continuous function*, *and let* $\varphi \in C^{2}([\alpha , \beta ])$, *where the image of* *g* *is a subset of* $[\alpha , \beta ]$. *Let* $\lambda :[a,b]\rightarrow \mathbb{R}$ *be a continuous function or a function of bounded variation such that* $\lambda (a)\neq \lambda (b)$ *and* $\overline{g}\in [\alpha , \beta ]$. *Let the functions* $G_{k}: [\alpha , \beta ]\times [\alpha , \beta ]\rightarrow \mathbb{R}$ ($k=0,1,2,3,4$) *be as defined in* (1)*–*(5). *Let* $x_{0} \in [a,b]$ *be arbitrarily chosen*, *and let* $g(x_{0})=c$.

*If for any* $k\in \{0,1,2,3,4\}$, *inequality* (14) *or the the reverse inequality in* (14) *holds for all* $s\in [\alpha , \beta ]$, *then*

21 $$ \biggl\vert \frac{\int_{a}^{b} \varphi ( g(x) ) \,d\lambda (x)}{\int _{a}^{b} \,d\lambda (x)}-\varphi ( \overline{g} ) \biggr\vert \leq \frac{1}{2} \cdot \bigl\Vert \varphi '' \bigr\Vert _{\infty }\cdot \biggl[ \biggl\vert \frac{\int_{a}^{b} ( g(x)-c ) ^{2} \,d\lambda (x)}{\int _{a}^{b} \,d\lambda (x)} \biggr\vert + \bigl\vert ( \overline{g}-c ) ^{2} \bigr\vert \biggr] . $$

### Proof

Let $x_{0} \in [a,b]$ be arbitrarily chosen, and let $g(x_{0})=c$. We have:

22 $$ \frac{\int_{a}^{b} ( g(x)- g(x_{0}) ) ^{2} \,d\lambda (x)}{\int _{a}^{b} \,d\lambda (x)} - \bigl( \overline{g}- g(x_{0}) \bigr) ^{2}=\frac{ \int_{a}^{b} ( g(x) ) ^{2} \,d\lambda (x)}{\int_{a}^{b} \,d\lambda (x)} - ( \overline{g} ) ^{2}. $$

Under the prepositions of the previous corollary, applying (22) in (15) and then using the triangle inequality, we get:

$$\begin{aligned} \biggl\vert \frac{\int_{a}^{b} \varphi ( g(x) ) \,d\lambda (x)}{\int _{a}^{b} \,d\lambda (x)}-\varphi ( \overline{g} ) \biggr\vert & \leq \frac{1}{2} \cdot \bigl\Vert \varphi '' \bigr\Vert _{\infty }\cdot \biggl\vert \frac{ \int_{a}^{b} ( g(x)-g(x_{0}) ) ^{2} \,d\lambda (x)}{\int_{a} ^{b} \,d\lambda (x)}- \bigl( \overline{g}-g(x_{0}) \bigr) ^{2} \biggr\vert \\ & \leq \frac{1}{2} \cdot \bigl\Vert \varphi '' \bigr\Vert _{\infty }\cdot \biggl[ \biggl\vert \frac{\int_{a}^{b} ( g(x)-c ) ^{2} \,d\lambda (x)}{ \int_{a}^{b} \,d\lambda (x)} \biggr\vert + \bigl\vert ( \overline{g}-c ) ^{2} \bigr\vert \biggr] . \end{aligned}$$

 □

## Discrete case

Discrete Jensen’s inequality states that

23 $$ \varphi \Biggl( \frac{1}{R_{n}}\sum _{i=1}^{n} r_{i} x_{i} \Biggr) \leq \frac{1}{R _{n}} \sum_{i=1}^{n} r_{i} \varphi (x_{i}) $$

for a convex function $\varphi : I \to \mathbf{R}$, $I \subseteq \mathbf{R}$, an *n*-tuple $\mathbf{x}= (x_{1},\ldots,x_{n})$ ($n\geq 2$), and a nonnegative *n*-tuple $\mathbf{r}= (r_{1},\ldots,r_{n})$ such that $\sum_{i=1}^{n} r_{i}>0$.

In [16] and [17], we have a generalization of that result. It is allowed that $r_{i}$ can also be negative with the sum different from 0, but there is given an additional condition on $r_{i}$, $x_{i}$ in terms of the Green functions $G_{k}: [\alpha , \beta ]\times [\alpha , \beta ]\rightarrow \mathbb{R}$ defined in (1)–(5).

To simplify the notation, we denote $R_{n}=\sum_{i=1}^{n} r_{i}$ and $\overline{x}=\frac{1}{R_{n}}\sum_{i=1}^{n} r_{i} x_{i}$.

As we already know (from Lemma 1) how to represent every function $\varphi \in C^{2}[\alpha , \beta ]$ in an adequate form using the previously defined functions $G_{k}$ ($k=0,1,2,3,4$), by some calculation it is easy to show that

24 $$ \frac{1}{R_{n}} \sum_{i=1}^{n} r_{i} \varphi (x_{i}) - \varphi ( \overline{x}) = \int_{\alpha }^{\beta } \Biggl( \frac{1}{R_{n}} \sum _{i=1}^{n} r_{i} G_{k}(x_{i},s)- G_{k}(\overline{x},s) \Biggr) \varphi ''(s)\,ds. $$

Similarly to the integral case, applying the Hölder inequality to (24), we get the following result.

### Theorem 2

*Let* $x_{i}\in [a,b]\subseteq [\alpha ,\beta ]$ *and* $r_{i} \in \mathbb{R}$ ($i=1,\ldots,n$) *be such that* $R_{n}\neq 0$ *and* $\overline{x}\in [\alpha ,\beta ]$, *and let* $\varphi \in C^{2}[\alpha , \beta ]$. *Let the functions* $G_{k}: [\alpha , \beta ]\times [\alpha , \beta ]\rightarrow \mathbb{R}$ ($k=0,1,2,3,4$) *be as defined in* (1)*–*(5). *Furthermore*, *let* $p,q\in \mathbb{R}$, $1\leq p,q\leq \infty $, *be such that* $\frac{1}{p}+\frac{1}{q}=1$. *Then*

25 $$ \Biggl\vert \frac{1}{R_{n}} \sum _{i=1}^{n} r_{i} \varphi (x_{i}) - \varphi (\overline{x}) \Biggr\vert \leq Q \cdot \bigl\Vert \varphi '' \bigr\Vert _{p}, $$

*where*

26 $$ Q= \textstyle\begin{cases} [ \int_{\alpha }^{\beta } \vert \frac{1}{R_{n}} \sum_{i=1}^{n} r _{i} G_{k}(x_{i},s)- G_{k}(\overline{x},s) \vert ^{q} \,ds ] ^{ \frac{1}{q}} & \textit{for } q \neq \infty ; \\ \sup_{s\in [\alpha ,\beta ]} \{ \vert \frac{1}{R_{n}} \sum_{i=1} ^{n} r_{i} G_{k}(x_{i},s)- G_{k}(\overline{x},s) \vert \} & \textit{for } q = \infty . \end{cases} $$

Set $q=1$, $p=\infty $. If the positivity of the term $\frac{1}{R_{n}} \sum_{i=1} ^{n} r_{i} G_{k}(x_{i},s)- G_{k}(\overline{x},s)$ for all $s\in [\alpha ,\beta ]$ does not change, then we can calculate *Q* and we get the following result.

### Corollary 3

*Let* $x_{i}\in [a,b]\subseteq [\alpha ,\beta ]$ *and* $r_{i} \in \mathbb{R}$ ($i=1,\ldots,n$), *be such that* $R_{n}\neq 0$ *and* $\overline{x}\in [\alpha ,\beta ]$, *and let* $\varphi \in C^{2}[\alpha , \beta ]$. *Let the functions* $G_{k}: [\alpha , \beta ]\times [\alpha , \beta ]\rightarrow \mathbb{R}$ ($k=0,1,2,3,4$) *be as defined in* (1)*–*(5). *If for any* $k\in \{0,1,2,3,4\}$, *the inequality*

27 $$ \frac{1}{R_{n}} \sum_{i=1}^{n} r_{i} G_{k}(x_{i},s)- G_{k}( \overline{x},s)\geq 0 $$

*or the reverse inequality in* (27) *holds for all* $s\in [\alpha , \beta ]$, *then*

28 $$ \Biggl\vert \frac{1}{R_{n}} \sum _{i=1}^{n} r_{i} \varphi (x_{i}) - \varphi (\overline{x}) \Biggr\vert \leq \frac{1}{2} \cdot \bigl\Vert \varphi '' \bigr\Vert _{\infty }\cdot \Biggl\vert \frac{1}{R_{n}} \sum _{i=1}^{n} r_{i} x_{i}^{2} - \overline{x}^{2} \Biggr\vert . $$

Let $c \in [a,b]\subseteq [\alpha ,\beta ]$ be arbitrarily chosen. Then

29 $$ \frac{1}{R_{n}} \sum_{i=1}^{n} r_{i} (x_{i}-c)^{2} - (\overline{x}-c)^{2}= \frac{1}{R _{n}} \sum_{i=1}^{n} r_{i} x_{i}^{2} - \overline{x}^{2}, $$

and we have the following result.

### Corollary 4

*Let* $x_{i}\in [a,b]\subseteq [\alpha ,\beta ]$ *and* $r_{i} \in \mathbb{R}$ ($i=1,\ldots,n$) *be such that* $R_{n}\neq 0$ *and* $\overline{x}\in [\alpha ,\beta ]$, *and let* $\varphi \in C^{2}[\alpha , \beta ]$. *Let the functions* $G_{k}: [\alpha , \beta ]\times [\alpha , \beta ]\rightarrow \mathbb{R}$ ($k=0,1,2,3,4$) *be as defined in* (1)*–*(5). *Let* $c \in [a,b]\subseteq [\alpha , \beta ]$ *be arbitrarily chosen*.

*If for any* $k\in \{0,1,2,3,4\}$, *inequality* (27) *or the reverse inequality in* (27) *holds for all* $s\in [ \alpha , \beta ]$, *then*

30 $$ \Biggl\vert \frac{1}{R_{n}} \sum _{i=1}^{n} r_{i} \varphi (x_{i}) - \varphi (\overline{x}) \Biggr\vert \leq \frac{1}{2} \cdot \bigl\Vert \varphi '' \bigr\Vert _{\infty }\cdot \Biggl[ \Biggl\vert \frac{1}{R_{n}} \sum _{i=1}^{n} r_{i} (x _{i}-c)^{2} \Biggr\vert + \bigl\vert (\overline{x}-c)^{2} \bigr\vert \Biggr] . $$

## Some applications

### Applications to Csiszár *f*-divergence

Divergences between probability distributions have been introduced to measure the difference between them. A lot of different types of divergences exist, for example, the *f*-divergence, Rényi divergence, Jensen–Shannon divergence, and so on (see, e.g., [8] and [18]). There are numerous applications of divergences in many fields: anthropology and genetic, economics, ecological studies, music, signal processing, and pattern recognition.

The Jensen inequality plays an important role in obtaining inequalities for divergences between probability distributions, and there are many papers dealing with inequalities for divergences and entropies (see, e.g., [7, 9, 12]).

In this section, we give some applications of our results, and we first introduce the basic notions.

Csiszár [3, 4] defined the *f*-divergence functional as follows.

#### Definition 1

Let $f:[ 0,\infty \rangle \rightarrow [ 0,\infty \rangle $ be a convex function, and let $\mathbf{p}:= ( p_{1},\ldots,p_{n} ) $ and $\mathbf{q}:= ( q_{1},\ldots,q_{n} ) $ be positive probability distributions. The *f*-divergence functional is

$$ D_{f}(\mathbf{p},\mathbf{q}):={ \sum_{i=1}^{n}} q_{i}f \biggl( \frac{p_{i}}{q_{i}} \biggr) . $$

The undefined expressions can be interpreted by

$$ f ( 0 ) :=\lim_{t\rightarrow 0+}f ( t ) ; \quad\quad 0f \biggl( \frac{0}{0} \biggr) :=0;\quad\quad 0f \biggl( \frac{a}{0} \biggr) := \lim_{t\rightarrow 0+}tf \biggl( \frac{a}{t} \biggr) ,\quad a>0. $$

This definition of the *f*-divergence functional can be generalized for a function $f:I\rightarrow \mathbb{R}$, $I\subset \mathbb{R}$, where $\frac{p_{i}}{q_{i}}\in I$ for $i=1,\ldots,n$, as follows (see also [7]).

#### Definition 2

Let $I\subset \mathbb{R}$ be an interval, and let $f:I\rightarrow \mathbb{R}$ be a function. Let $\mathbf{p}:= ( p_{1},\ldots,p _{n} ) \in \mathbb{R}^{n}$ and $\mathbf{q}:= ( q_{1},\ldots,q_{n} ) \in \langle 0,\infty \rangle^{n}$ be such that

$$ \frac{p_{i}}{q_{i}}\in I,\quad i=1,\ldots,n. $$

Then let

$$ \hat{D}_{f}(\mathbf{p},\mathbf{q}):={ \sum _{i=1}^{n}} q_{i}f \biggl( \frac{p_{i}}{q_{i}} \biggr) . $$

Now we apply Theorem 2 to $\hat{D}_{f}(\mathbf{p},\mathbf{q})$ and get the following result.

#### Theorem 3

*Let* $\mathbf{p}:= ( p_{1},\ldots,p_{n} ) \in \mathbb{R}^{n}$, *and* $\mathbf{q}:= ( q_{1},\ldots,q_{n} ) \in \langle 0, \infty \rangle^{n}$ *be such that*

$$ \frac{p_{i}}{q_{i}}\in [a,b]\subseteq [\alpha ,\beta ] \quad \textit{for } i=1,\ldots,n \quad \textit{and}\quad \frac{\sum_{i=1}^{n} p_{i}}{\sum_{i=1}^{n} q _{i}}\in [\alpha ,\beta ]. $$

*Let the functions* $G_{k}: [\alpha , \beta ]\times [\alpha , \beta ] \rightarrow \mathbb{R}$ ($k=0,1,2,3,4$) *be as defined in* (1)*–*(5). *Furthermore*, *let* $p,q\in \mathbb{R}$, $1\leq p,q\leq \infty $, *be such that* $\frac{1}{p}+\frac{1}{q}=1$.

- *If* $f: [\alpha , \beta ]\rightarrow \mathbb{R}$, $f \in C^{2}[\alpha , \beta ]$, *then*
  31 $$ \biggl\vert \frac{1}{\sum_{i=1}^{n} q_{i}} \hat{D}_{f}( \mathbf{p}, \mathbf{q}) - f \biggl( \frac{\sum_{i=1}^{n} p_{i}}{\sum_{i=1}^{n} q_{i}} \biggr) \biggr\vert \leq Q \cdot \bigl\Vert f'' \bigr\Vert _{p}, $$
  *where*
  $$ Q= \textstyle\begin{cases} [ \int_{\alpha }^{\beta } \vert \frac{1}{\sum_{i=1}^{n} q_{i}} \hat{D}_{G_{k} (\cdot ,s)}(\mathbf{p},\mathbf{q})- G_{k} ( \frac{ \sum_{i=1}^{n} p_{i}}{\sum_{i=1}^{n} q_{i}},s ) \vert ^{q} \,ds ] ^{\frac{1}{q}} & \textit{for } q \neq \infty , \\ \sup_{s\in [\alpha ,\beta ]} \{ \vert \frac{1}{\sum_{i=1}^{n} q _{i}} \hat{D}_{G_{k} (\cdot ,s)}(\mathbf{p},\mathbf{q})- G_{k} ( \frac{ \sum_{i=1}^{n} p_{i}}{\sum_{i=1}^{n} q_{i}},s ) \vert \} & \textit{for } q = \infty . \end{cases} $$
- *If* $\mathit{id} \cdot f \in C^{2}[\alpha , \beta ]$, *then*
  32 $$ \biggl\vert \frac{1}{\sum_{i=1}^{n} q_{i}} \hat{D}_{\mathit{id} \cdot f}( \mathbf{p}, \mathbf{q}) - \frac{\sum_{i=1}^{n} p_{i}}{\sum_{i=1}^{n} q_{i}} f \biggl( \frac{\sum_{i=1}^{n} p_{i}}{\sum_{i=1}^{n} q_{i}} \biggr) \biggr\vert \leq Q \cdot \bigl\Vert (\mathit{id}\cdot f)'' \bigr\Vert _{p}, $$
  *where* *id* *is the identity function*, $\hat{D}_{\mathit{id} \cdot f}(\mathbf{p}, \mathbf{q}) = { \sum_{i=1}^{n}} p_{i}f ( \frac{p_{i}}{q_{i}} ) $, *and*
  $$ Q= \textstyle\begin{cases} [ \int_{\alpha }^{\beta } \vert \frac{1}{\sum_{i=1}^{n} q_{i}} \hat{D}_{G_{k} (\cdot ,s)}(\mathbf{p},\mathbf{q})- G_{k} ( \frac{ \sum_{i=1}^{n} p_{i}}{\sum_{i=1}^{n} q_{i}},s ) \vert ^{q} \,ds ] ^{\frac{1}{q}} & \textit{for } q \neq \infty , \\ \sup_{s\in [\alpha ,\beta ]} \{ \vert \frac{1}{\sum_{i=1}^{n} q _{i}} \hat{D}_{G_{k} (\cdot ,s)}(\mathbf{p},\mathbf{q})- G_{k} ( \frac{ \sum_{i=1}^{n} p_{i}}{\sum_{i=1}^{n} q_{i}},s ) \vert \} & \textit{for } q = \infty . \end{cases} $$

#### Proof

(a) The result follows directly from Theorem 2 by substitution $\varphi :=f$,

$$ r_{i}:=\frac{q_{i}}{{ \sum_{i=1}^{n}} q_{i}},\quad\quad x_{i}:= \frac{p_{i}}{q_{i}},\quad i=1,\ldots,n. $$

(b) The result follows from (a) by substitution $f:=\mathit{id} \cdot f$. □

Let us mention two particular cases of the previous result.

The first one corresponds to the entropy of a positive probability distribution.

#### Definition 3

The Shannon entropy of a positive probability distribution $\mathbf{p}:= ( p_{1},\ldots,p_{n} ) $ is defined by

$$ H ( \mathbf{p} ) :=- { \sum_{i=1}^{n}} p_{i}\log ( p_{i} ) . $$

#### Corollary 5

*Let* $[\alpha , \beta ]\subseteq \langle 0,\infty \rangle $, *and let* $\mathbf{q}:= ( q_{1},\ldots,q_{n} ) \in \langle 0,\infty \rangle^{n}$ *be such that*

$$ \frac{1}{q_{i}}\in [a,b]\subseteq [\alpha ,\beta ] \quad \textit{for } i=1,\ldots,n \quad \textit{and}\quad \frac{n}{\sum_{i=1}^{n} q_{i}}\in [\alpha , \beta ]. $$

*Let the functions* $G_{k}: [\alpha , \beta ]\times [\alpha , \beta ] \rightarrow \mathbb{R}$ ($k=0,1,2,3,4$) *be as defined in* (1)*–*(5). *Furthermore*, *let* $p,q\in \mathbb{R}$, $1\leq p,q\leq \infty $, *be such that* $\frac{1}{p}+\frac{1}{q}=1$. *Then*

33 $$ \biggl\vert \frac{1}{\sum_{i=1}^{n} q_{i}} H ( \mathbf{q} ) - \log \biggl( \frac{n}{\sum_{i=1}^{n} q_{i}} \biggr) \biggr\vert \leq Q \cdot \bigl\Vert \log '' \bigr\Vert _{p}, $$

*where*

$$ Q= \textstyle\begin{cases} [ \int_{\alpha }^{\beta } \vert \frac{1}{\sum_{i=1}^{n} q_{i}} \sum_{i=1}^{n} q_{i} G_{k} ( \frac{1}{q_{i}},s ) - G_{k} ( \frac{n}{ \sum_{i=1}^{n} q_{i}},s ) \vert ^{q} \,ds ] ^{\frac{1}{q}} & \textit{for } q \neq \infty , \\ \sup_{s\in [\alpha ,\beta ]} \{ \vert \frac{1}{\sum_{i=1}^{n} q _{i}} \sum_{i=1}^{n} q_{i} G_{k} ( \frac{1}{q_{i}},s ) - G_{k} ( \frac{n}{\sum_{i=1}^{n} q_{i}},s ) \vert \} & \textit{for } q = \infty . \end{cases} $$

#### Proof

The result follows directly from Theorem 3(a) by substitution $f:=\log $ and $\mathbf{p}:= ( 1,\ldots,1 ) $. □

The second one corresponds to the relative entropy or Kullback–Leibler divergence between two probability distributions.

#### Definition 4

The Kullback–Leibler divergence between two positive probability distributions $\mathbf{p}:= ( p_{1},\ldots,p_{n} ) $ and $\mathbf{q}:= ( q_{1},\ldots,q_{n} ) $ is defined by

$$ D ( \mathbf{p}\shortparallel \mathbf{q} ) :={ \sum_{i=1}^{n}} p_{i}\log \biggl( \frac{p_{i}}{q_{i}} \biggr) . $$

#### Corollary 6

*Let* $[\alpha , \beta ]\subseteq \langle 0,\infty \rangle $, *and let* $\mathbf{p}:= ( p_{1},\ldots,p_{n} ) \in \mathbb{R}^{n}$ *and* $\mathbf{q}:= ( q_{1},\ldots,q_{n} ) \in \langle 0,\infty \rangle^{n}$ *be such that*

$$ \frac{p_{i}}{q_{i}}\in [a,b]\subseteq [\alpha ,\beta ] \quad \textit{for } i=1,\ldots,n \quad \textit{and}\quad \frac{\sum_{i=1}^{n} p_{i}}{\sum_{i=1}^{n} q _{i}}\in [\alpha ,\beta ]. $$

*Let the functions* $G_{k}: [\alpha , \beta ]\times [\alpha , \beta ] \rightarrow \mathbb{R}$ ($k=0,1,2,3,4$) *be as defined in* (1)*–*(5). *Furthermore*, *let* $p,q\in \mathbb{R}$, $1\leq p,q\leq \infty $, *be such that* $\frac{1}{p}+\frac{1}{q}=1$.

*Then*

$$ \biggl\vert \frac{1}{\sum_{i=1}^{n} q_{i}} D ( \mathbf{p}\shortparallel \mathbf{q} ) - \frac{\sum_{i=1}^{n} p_{i}}{\sum_{i=1}^{n} q_{i}} \log \biggl( \frac{\sum_{i=1}^{n} p_{i}}{\sum_{i=1}^{n} q_{i}} \biggr) \biggr\vert \leq Q \cdot \bigl\Vert (\mathit{id}\cdot \log )'' \bigr\Vert _{p}, $$

*where* *id* *is the identity function*, *and*

$$ Q= \textstyle\begin{cases} [ \int_{\alpha }^{\beta } \vert \frac{1}{\sum_{i=1}^{n} q_{i}} \sum_{i=1}^{n}q_{i}G_{k} ( \frac{p_{i}}{q_{i}},s ) - G_{k} ( \frac{\sum_{i=1}^{n} p_{i}}{\sum_{i=1}^{n} q_{i}},s ) \vert ^{q} \,ds ] ^{\frac{1}{q}} & \textit{for } q \neq \infty , \\ \sup_{s\in [\alpha ,\beta ]} \{ \vert \frac{1}{\sum_{i=1}^{n} q _{i}} \sum_{i=1}^{n}q_{i}G_{k} ( \frac{p_{i}}{q_{i}},s ) - G _{k} ( \frac{\sum_{i=1}^{n} p_{i}}{\sum_{i=1}^{n} q_{i}},s ) \vert \} &\textit{for } q = \infty . \end{cases} $$

#### Proof

The result follows from Theorem 3(b) by substitution $f:=\log $. □

### Applications to Zipf–Mandelbrot law

The forthcoming results deal with the so called Zipf–Mandelbrot law.

George Kingsley Zipf (1902–1950) was a linguist who investigated the frequencies of different words in the text. The Zipf law is one of the basic laws in information science, bibliometrics, and linguistics (see [5]). In certain fields, like economics and econometrics, this distribution is known as Pareto’s law. There it analyzes the distribution of the wealthiest members of the community (see [5], p. 125). Though, in the mathematical sense, these two laws are the same; the only difference is that they are applied in different contexts (see [6], p. 294). The same kind of distribution can be also found in other scientific disciplines, such as physics, biology, earth and planetary sciences, computer science, demography, and social sciences. For more information, we refer [15].

The mathematician Benoit Mandelbrot (1924–2010) introduced a more general model of this law (see [11]). The new Zipf–Mandelbrot law has many different applications, for example, in information sciences [6], linguistics [13], ecology [14], music [10], and so on.

#### Definition 5

([7])

The Zipf–Mandelbrot law is a discrete probability distribution, depending on three parameters $N\in \{ 1,2,\ldots \} $, $t\in [0,\infty \rangle $, and $v>0$ and defined by

$$ f ( i;N,t,v ) :=\frac{1}{ ( i+t ) ^{v}H_{N,t,v}}, \quad i=1,\ldots,N, $$

where

$$ H_{N,t,v}:= { \sum_{j=1}^{N}}\frac{1}{ ( j+t ) ^{v}}. $$

When $t=0$, then the Zipf–Mandelbrot law becomes the Zipf law.

Now, we will apply our results for distributions given in Theorem 3 to the Zipf–Mandelbrot law, a sort of discrete probability distributions.

#### Corollary 7

*Let* $\mathbf{p_{1}}$, $\mathbf{p_{2}}$ *be two Zipf–Mandelbrot laws with parameters* $N\in \{ 1,2,\ldots \} $, $t_{1}, t_{2}\in [0, \infty \rangle $, *and* $v_{1}$, $v_{2}>0$, *respectively*, *such that*

$$\begin{aligned}& \frac{ ( i+t_{2} ) ^{v_{2}}H_{N,t_{2},v_{2}}}{ ( i+t_{1} ) ^{v_{1}}H_{N,t_{1},v_{1}}}\in [a,b]\subseteq [\alpha , \beta ] \quad \textit{for } i=1, \ldots,N \\& \textit{and} \quad \frac{A_{1}}{A_{2}}\in [\alpha ,\beta ], \quad \textit{where } A_{j}:=\sum_{i=1}^{N} \frac{1}{ ( i+t_{j} ) ^{v_{j}}H_{N,t _{j},v_{j}}} \ (j=1,2). \end{aligned}$$

*Let the functions* $G_{k}: [\alpha , \beta ]\times [\alpha , \beta ] \rightarrow \mathbb{R}$ ($k=0,1,2,3,4$) *be as defined in* (1)*–*(5). *Furthermore*, *let* $p,q\in \mathbb{R}$, $1\leq p,q\leq \infty $, *be such that* $\frac{1}{p}+\frac{1}{q}=1$.

- *If* $f: [\alpha , \beta ]\rightarrow \mathbb{R}$, $f \in C^{2} ( [\alpha , \beta ] ) $, *then*
  $$ \biggl\vert \frac{1}{A_{2}} \hat{D}_{f}(\mathbf{p_{1}}, \mathbf{p_{2}}) - f \biggl( \frac{A_{1}}{A_{2}} \biggr) \biggr\vert \leq Q \cdot \bigl\Vert f'' \bigr\Vert _{p}, $$
  *and*
- *if* $\mathit{id} \cdot f \in C^{2}[\alpha , \beta ]$, *then*
  $$ \biggl\vert \frac{1}{A_{2}} \hat{D}_{\mathit{id} \cdot f}(\mathbf{p_{1}}, \mathbf{p_{2}}) - \frac{A_{1}}{A_{2}} f \biggl( \frac{A_{1}}{A_{2}} \biggr) \biggr\vert \leq Q \cdot \bigl\Vert (\mathit{id} \cdot f)'' \bigr\Vert _{p}, $$
  *where* *id* *is the identity function*, *and*
  $$ Q= \textstyle\begin{cases} [ \int_{\alpha }^{\beta } \vert \frac{1}{A_{2}} \hat{D}_{G_{k} ( \cdot ,s)}(\mathbf{p_{1}},\mathbf{p_{2}})- G_{k} ( \frac{A_{1}}{A _{2}},s ) \vert ^{q} \,ds ] ^{\frac{1}{q}} & \textit{for } q \neq \infty , \\ \sup_{s\in [\alpha ,\beta ]} \{ \vert \frac{1}{A_{2}} \hat{D} _{G_{k} (\cdot ,s)}(\mathbf{p_{1}},\mathbf{p_{2}})- G_{k} ( \frac{A _{1}}{A_{2}},s ) \vert \} & \textit{for } q = \infty . \end{cases} $$

Although it is a particular case of the result just given, here we also present a result for the Shannon entropy.

#### Corollary 8

*Let* $[\alpha , \beta ]\subseteq \langle 0,\infty \rangle $, *let* **q** *be the Zipf–Mandelbrot law as defined in Definition *5 *such that*

$$\begin{aligned}& ( i+t ) ^{v}H_{N,t,v}\in [a,b]\subseteq [\alpha ,\beta ] \quad \textit{for } i=1,\ldots,N \\& \textit{and}\quad \frac{N}{B}\in [\alpha ,\beta ], \quad \textit{where } B := \sum _{i=1}^{N} \frac{1}{ ( i+t ) ^{v}H_{N,t,v}}. \end{aligned}$$

*Let the functions* $G_{k}: [\alpha , \beta ]\times [\alpha , \beta ] \rightarrow \mathbb{R}$ ($k=0,1,2,3,4$) *be as defined in* (1)*–*(5). *Furthermore*, *let* $p,q\in \mathbb{R}$, $1\leq p,q\leq \infty $, *be such that* $\frac{1}{p}+\frac{1}{q}=1$.

*Then*

$$ \biggl\vert \frac{1}{B} H ( \mathbf{q} ) - \log \biggl( \frac{N}{B} \biggr) \biggr\vert \leq Q \cdot \bigl\Vert \log '' \bigr\Vert _{p}, $$

*where*

$$ Q= \textstyle\begin{cases} [ \int_{\alpha }^{\beta } \vert \frac{1}{B} \sum_{i=1}^{N} \frac{1}{ ( i+t ) ^{v}H_{N,t,v}} G_{k} ( ( i+t ) ^{v}H _{N,t,v};s ) - G_{k} ( \frac{N}{B},s ) \vert ^{q} \,ds ] ^{\frac{1}{q}} &\textit{for } q \neq \infty , \\ \sup_{s\in [\alpha ,\beta ]} \{ \vert \frac{1}{B} \sum_{i=1}^{N} \frac{1}{ ( i+t ) ^{v}H_{N,t,v}} G_{k} ( ( i+t ) ^{v}H_{N,t,v};s ) - G_{k} ( \frac{N}{B},s ) \vert \} &\textit{for } q = \infty . \end{cases} $$
